# Growth Modification of Developing Class II Division 1 Malocclusion Using Myofunctional Appliances

**DOI:** 10.1155/2023/8201195

**Published:** 2023-09-19

**Authors:** Ananto Ali Alhasyimi, Iman Syahfik

**Affiliations:** ^1^Department of Orthodontics, Faculty of Dentistry, Universitas Gadjah Mada, Yogyakarta, Indonesia; ^2^Private Dental Clinic, Depok, Indonesia

## Abstract

*Background and Overview*. Interceptive orthodontics is a phase of orthodontics that identifies and corrects potential malocclusions in the growing dentofacial complex. At this point, myofunctional appliances are a necessary component. A 9-year-old girl appeared with a Class II division 1, a 6 mm overjet, a 6 mm overbite, a V-shaped maxillary arch, as well as lip hypertonicity, and an overdeveloped maxilla. Myobrace® combines the dental alignment abilities of rigid and soft appliances. Its structure replicates a fixed appliance: the soft outer part acts as the orthodontic wire, whereas the inner hard part engages the teeth individually. After 12 months of treatment, facial photos and a cephalogram were taken, both of which demonstrated an enhancement to the patient's facial profile. *Conclusions and Practical Implications*. This case demonstrates that Myobrace® may be a simple and good choice to treat skeletal malocclusion with oral habit because of its greater compliance and favorable results. In certain instances, an early approach, taking into account patient preferences and compliance, is practical and should be considered in future treatment planning and research.

## 1. Introduction

The fundamental goals of orthodontics are to address patients' complaints, establish optimal functional outcomes, and promote favorable aesthetic outcomes [1]. The appropriate time to begin orthodontic treatment for patients with malocclusions has been the subject of much discussion among orthodontists. It is essential to the development of children and adolescents to maintain some measure of control over the growth of their dentition and occlusion. Early intervention is key in interceptive orthodontics, which focuses on correcting skeletal and dental malocclusions. Interceptive orthodontics refers to performing orthodontic therapy on young children to minimize or simplify later orthodontic treatment [2]. Detecting malocclusions in their earliest stages is advantageous. A simplified strategy is preferred for interceptive orthodontics in younger patients [3]. During the mixed dentition stage, one of the most common and serious dental issues that can arise is Class II malocclusion. This issue can affect a person's smile for their entire life, which requires early treatment to alleviate the severity of the malocclusion. Children with mixed dentition have greater growth potential. Utilizing the growth potential more effectively could result in more effective and stable results [4]. Orthopedic appliances produce a new musculoskeletal and functional environment for the face bones, which encourages alterations in either the mandible or the maxilla [5]. Functional appliances are a common orthodontic therapy for growing children to correct skeletal discrepancies [6]. Jasper jumper, activator, bionator, twin block, and forsus are functional appliances that accelerate mandibular growth. Even though therapy success is dependent on patient compliance, many patients reject these unpleasant functional devices. Few ordinary appliances can simulate muscle training. Muscle dysfunction must be eliminated for long-term therapy success [7, 8].

Orofacial myofunctional therapy is a rapidly expanding area of orthodontic treatment, which focuses on correcting facial muscular imbalances as well as teaching proper tongue postures, and reestablishing harmony between the lip, cheek muscles, and tongue. Additionally, it has been demonstrated that myofunctional therapy may influence changes in dental development. Prefabricated functional appliances have been shown to have a beneficial effect on children with Class II division 1 malocclusions [9, 10]. Myobrace® devices are prefabricated functional appliances, including features for tooth positioning and myofunctional exercise that are used in combination with myofunctional therapy—via the Myobrace® protocol—to address malocclusions in growing children [9]. Thus, this study aims to describe a mixed dentition malocclusion that was effectively treated using Myobrace® appliances.

## 2. Case Report and Case Management

**Figure 1:**
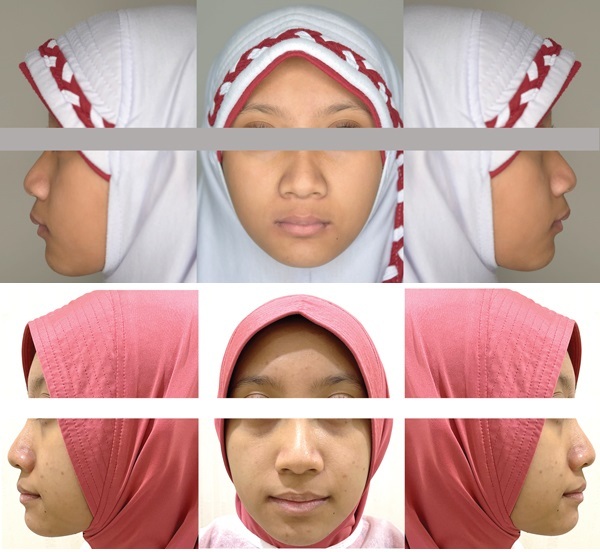
(a) Pre-treatment and (b) post-treatment extraoral photographs; frontal and lateral view profile view at rest, respectively.

**Figure 2:**
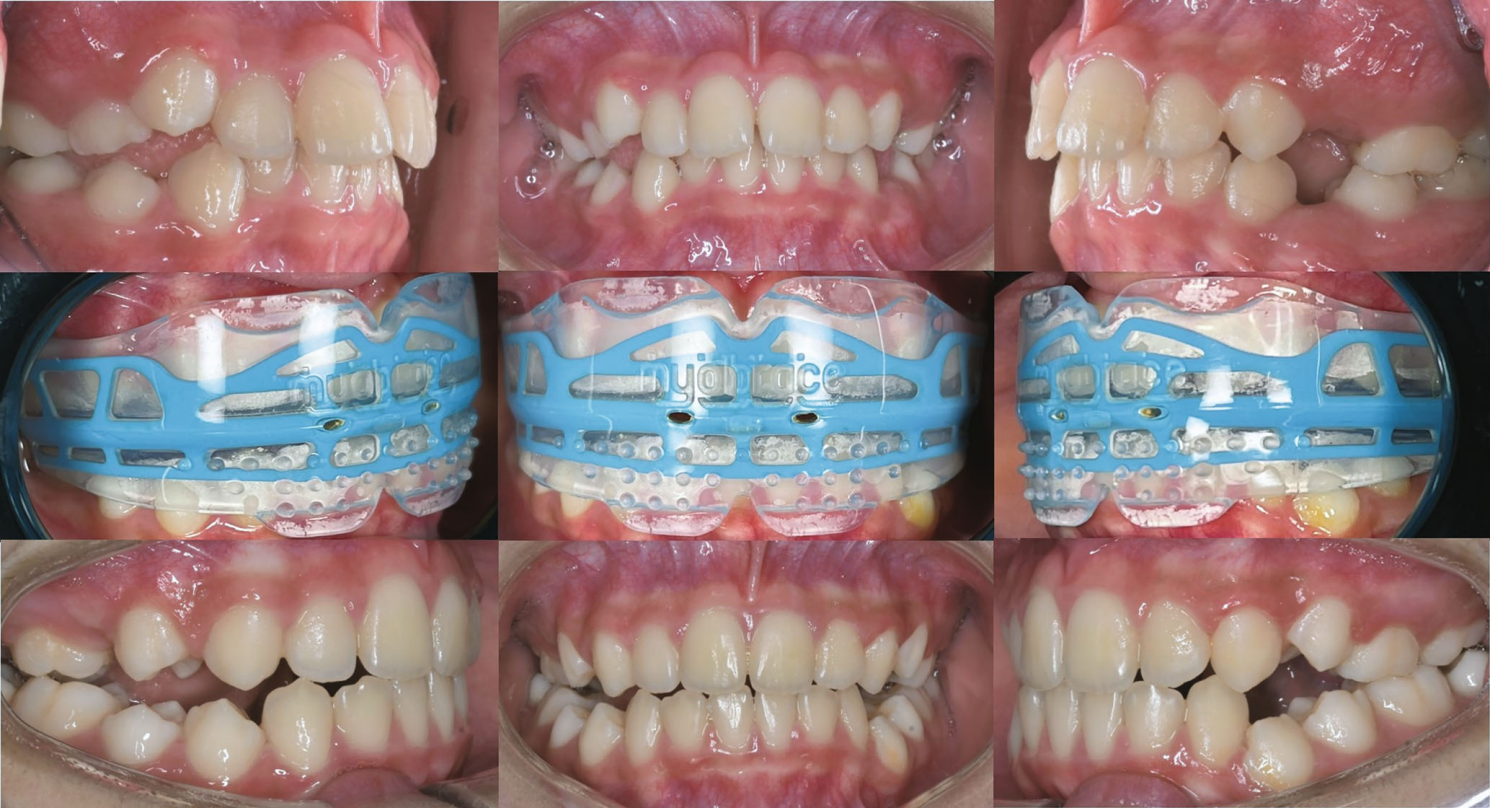
Pre- and post-treatment intraoral radiographs, before treatment (upper), during treatment (middle), and after treatment (lower).

**Figure 3:**
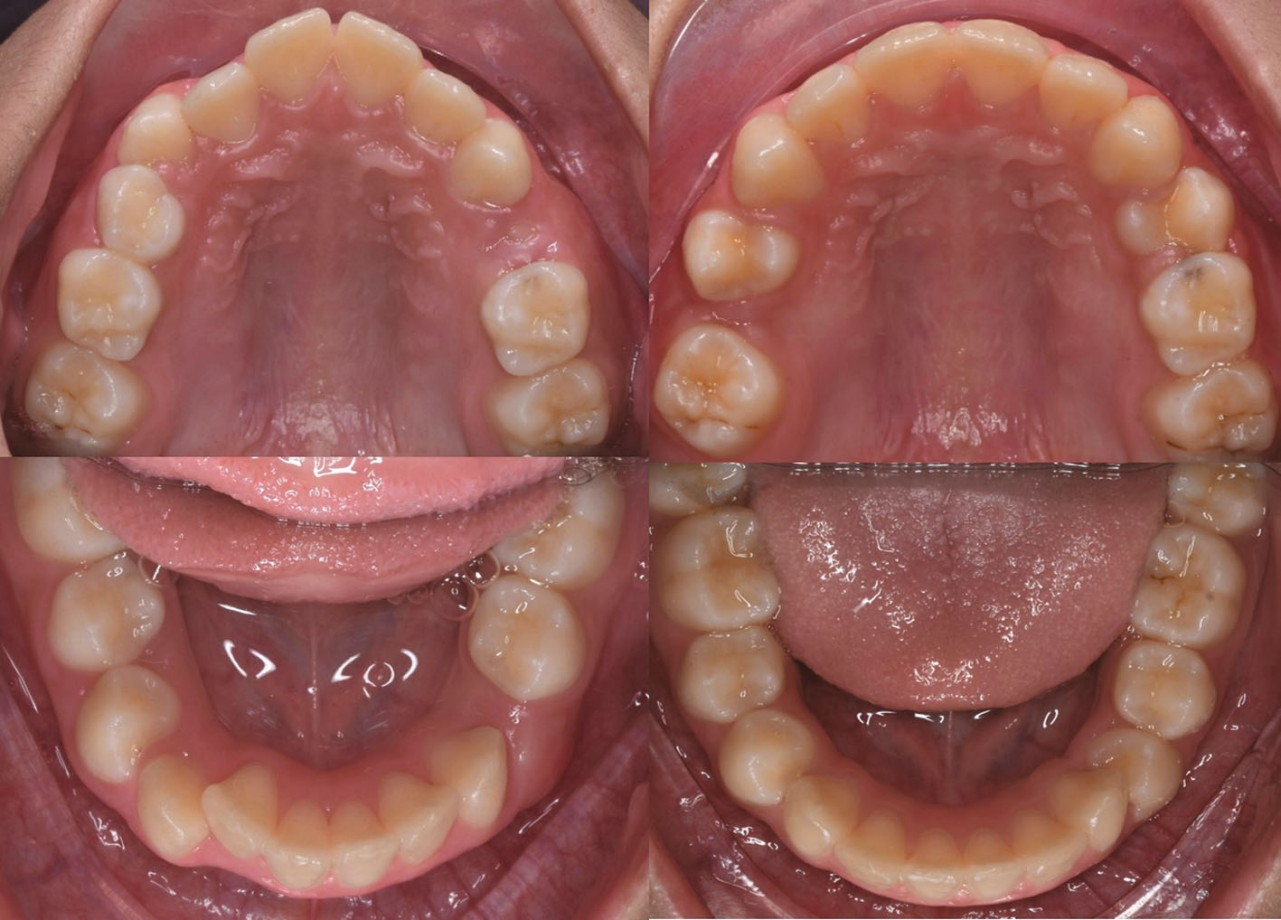
Pre- and post-treatment occlusal view, before (upper), and after treatment (lower).

**Figure 4:**
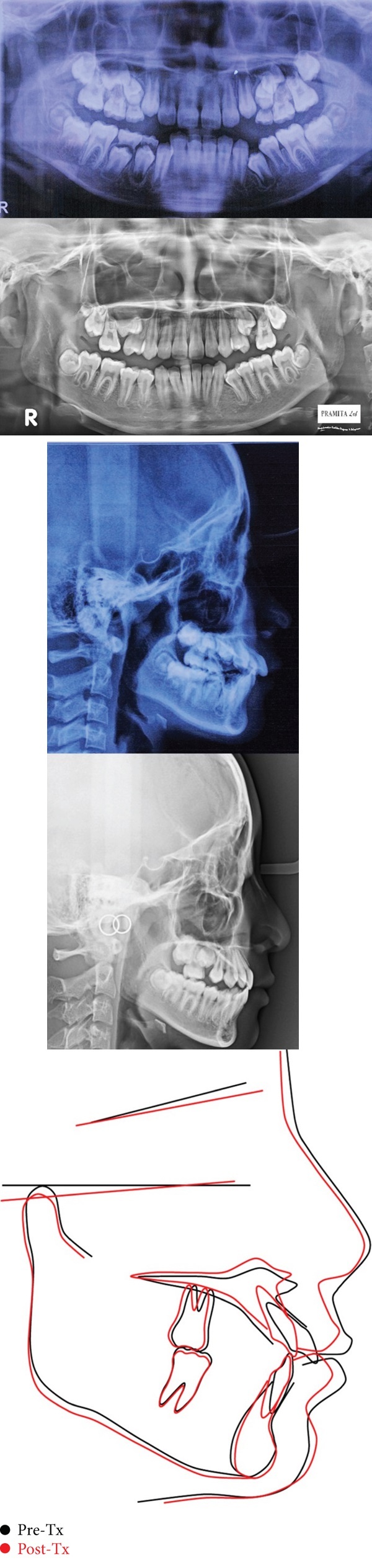
(a) Pre- and post-treatment panoramic radiographs, (b) lateral cephalometric, and (c) tracings of lateral cephalometric superimposed; black lines represent tracing before treatments, and red lines show the tracing after treatments.

A female patient of 9 years old presented to the dental clinic with the chief complaint of protrusive upper anterior teeth. The clinical examination revealed a convex profile with a protrusive maxilla, retrusive mandible, an increased lower anterior facial height, hypotonic upper lip, deep labiomental fold, and hyperactivity of the mentalis and buccinator muscles (Figure 1(a)). The prevalence of hyperactivity in the mentalis and buccinator muscles is frequently observed in individuals with lip incompetence or those exhibiting protrusion of the upper incisors. She tended to mouth breathing due to seasonal allergies, and lip sucking. Intraoral examination revealed Angle's Class II relationship bilaterally with a protrusion (large overjet up to 6 mm), deep overbite of 6 mm, and constricted maxillary dental arch (Figures 2 and 3). The teeth #55, #54, #65, and #75 were not replaced (Figure 3). Due to a prognathic maxilla and an increase in ANB Angle, pretreatment cephalometric data indicated a developing skeletal Class II (Table 1). The patient was in the prepubertal growth period/Cervico Vertebrae Maturation Index (CVMI) stage 1 [the inferior margins of the second, third, and fourth cervical vertebrae (C2, C3, and C4) exhibit a flat morphology. The shapes of the bodies of C3 and C4 vertebrae are trapezoidal, with their superior vertebral borders gradually narrowing from posterior to anterior] based on the supporting examination employing cephalometry (Figure 4(b), upper part/cephalometric before treatment).

The treatment objective was to enhance alveolar development, alleviate crowding, correct maxillo-mandibular relationship, and modify vertical growth patterns. The skeletal growth of the patient was seen to be in the prepubertal phase, specifically classified as CVMI stage 1. This suggests that there is a possibility for the skeletal growth to be altered towards Class I skeletal growth. Wearing the Myobrace® T1 appliance for one hour each day, plus overnight, was prescribed for the patient for the first six months, to initiate habit correction (training the patient to breathe through their nose rather than through their mouth, adapting their tongue to rest in the appropriate position, and swallowing correctly), followed by the T2 appliance for 4 months, which provides arch development. The Myobrace® T3 was the final appliance that the patient wore for 2 hours daytime plus overnight, for five months. This appliance gives additional space for erupting teeth.

After 12 months of treatment, anterior teeth positions, particularly the maxillary incisors, improved significantly. Inter-canine and inter-molar widths increased (upper inter-canine width from 30.82 to 35.16 mm; lower inter-canine width from 26.92 to 30.26 mm; upper inter-molar width from 48.67 to 49.48 mm; and lower inter-molar width from 44.08 to 45.06 mm; Figure 3). Profile improvement (Figure 1(b)), both overjet and overbite were reduced by four millimeters, and a relationship corresponding to Angle Class I was obtained. In addition, while the lips were brought together, there was less pressure on the orofacial muscles when they were at rest, and there was less hyperactivity in the buccinator and mentalis muscles when swallowing.

The cephalometric study (Figure 4; Table 1) revealed that the ANB angle was 2 (from 5°), and the Wits appraisal was 2.5 mm (from 6.5 mm). All of these exhibited the skeletal pattern transition from Class II to Class I. The lateral superimpositions revealed that the maxillary incisors had retracted approximately 5°, the mandibular incisors had proclined, the angle of convexity improved from 6° to 2°, the angle between the maxillary and mandibular planes increased from 26° to 28°, which resulted in a slight elevation of the lower facial height. No apical root resorption was evident, and root parallelism was good (Figure 4).

## 3. Discussion

Delaying orthodontic treatment until all permanent teeth have erupted, unfortunately, can cause irreversible damage to their teeth and overall health and development as a whole. Because poor oral habits are readily apparent before all of the permanent teeth erupt, this finding suggests that treatment for the underlying problems can begin significantly earlier than was previously believed. Mouth breathing, tongue thrusting, thumb, and lip sucking are all frequent bad habits in children. A chronic, long-term habit may create detrimental effects on the dentofacial structures [11]. Myofunctional therapy was successful in returning the tongue to its normal position at rest, and it demonstrated an improvement that was both better and faster in the tongue's resting posture [12]. The literature describes functional appliances as an effective strategy for correcting functional problems in the orofacial region. However, the success of treatment using functional appliances depends on two crucial factors: patient cooperation and appliance design. The former is contingent upon the dentist's ability to persuade the patient of the necessity of therapy and the detrimental consequences of oral dysfunctions on the performance and health of the stomatognathic system. Thankfully, the latter issue has been addressed by the orthodontic community. The development of a prefabricated functional appliance that is designed to adapt to different age groups and, as a result, fix the functional issues found in young individuals. These devices cure malocclusions not by moving the teeth directly, but by correcting myofunctional changes, thereby allowing the teeth to occupy a more stable physiological position in the oral cavity [13]. Myobrace® is meant to rectify these improper myofunctional behaviors by instructing patients to breathe via nose instead of mouth, relax their tongue appropriately on the tip of the hard palate, swallow correctly, keep the lips together when not eating or speaking, and continue expanding their maxilla and mandible so that they develop naturally to their right way. This creates adequate space for the teeth, which enables the teeth to erupt in a proper position.

Myobrace® dramatically raised the axial inclination of the mandibular incisor to the mandibular plane (L1-MP), decreased the axial inclination of the maxillary incisor to the palatal plane, and increased interincisal angle, indicating an overjet reduction. The interincisal angle can only be reduced by proclinating either the mandibular or maxillary incisors, or both, and it must be adjusted so that the tip of the mandibular incisor occludes onto the cingulum of the maxillary incisor [14, 15]. Similar results were reported for the twin block appliance. The overjet reduction experienced by these devices may be attributable to the stronger action achieved by utilizing the resultant masticatory forces to redirect the arches toward a normal relationship [16]. The use of prefabricated myofunctional appliances to treat various types of malocclusion, particularly Class II malocclusion, has gained favor. According to the findings of a Cochrane review that was just recently brought up to date, a variety of different functional appliances were successful in reducing the prominence of the upper anterior teeth [17]. They treat dysfunctional orofacial muscle activity, tongue position, and airway volume to target the etiological factors that contribute to malocclusion, which ultimately results in more stable occlusion [18]. Overjet reduction also improves the maxillomandibular skeletal relationship in the sagittal dimension and increases the lower anterior facial height. The cephalometric analysis reveals a slight retrusion of the maxilla and mandibular protraction induced by the use of Myobrace®. When the mandible was positioned in an anterior direction, a reciprocal force was exerted in a posterior direction on the maxilla, limiting its anterior growth. This phenomenon is commonly referred to as the headgear effect. Even though point A is a deep alveolar point in the maxilla, it is also influenced by dentoalveolar alterations [19].

A properly developed maxilla, advancement of the mandible, an increase in the vertical dimension, and balanced soft tissue profile as evaluated by cephalometry were regarded to be direct effects of the appliance treatment. With Myobrace® therapy, the tongue is properly positioned in the upper jaw. This enables efficient breathing and swallowing patterns. During the growing phase, breathing through the nose causes a sequence of bone and muscle modifications. The appliance also retrains the oral muscles, which expand the jaws and straighten the teeth by exerting small forces. The tongue tag, guard, and elevators of the Myobrace® appliance reposition the tongue and prevent lip sucking. The expanded lip bumper inhibits powerful, hyperactive lip muscles, hence enhancing their tonicity.

This patient had a considerable rise in both the inter-canine and inter-molar widths. When abnormal oral muscles are retrained, the ensuing forces are directed toward the jaws, where they contribute to the enlargement of the dental arches and the proper alignment of irregular teeth. This is made possible by the Dynamicore with Frankel grid construction found in the Myobrace®, which contributes to the expansion and development of the jaws. The air spring enables active and moderate stimulation to be applied to the developing muscles of the face and jaw. Dynamicore is used as a wire and dental slots are used to lengthen the dental arch in T3 of the Myobrace® system, which is the final stage of the therapy. T3 must be used regularly to prevent the arch from shrinking, which occurs if the device is not utilized regularly. Unlike T1 and T2, T3 is primarily focused on dental alignment, yet it exhibits habits of correction comparable with those of T1 and T2.

Myobrace® was created to combine the dental alignment capability of stiff appliances with the qualities of soft and flexible ones. Its construction is consequently designed to resemble a fixed appliance: the outer soft portion functions as the orthodontic wire, whereas the inner hard portion engages the teeth individually, simulating the function of the brackets. This twofold structure suggests a higher level of acceptability and enhances patient compliance. Consequently, its usage is recommended for patients, particularly in the mixed dentition phase, because of its superior compliance and favorable results [20]. Compliance on the part of the patient is of the utmost importance for the achievement of desirable results during orthodontic treatment using removable devices [21]. Cooperation from patients and motivation on their part are necessary for achieving the best possible outcome.

**Figure 5:**
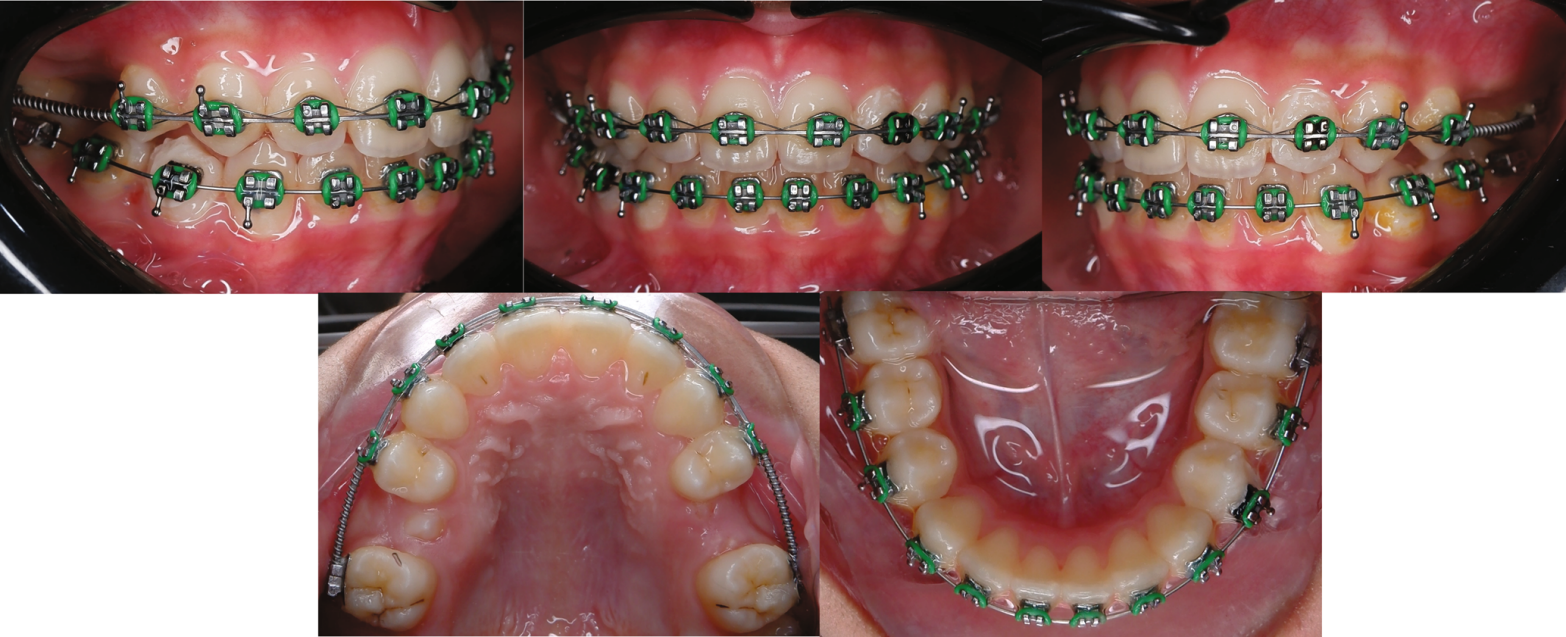
The progress of the treatment for 3 months with fixed orthodontic appliances.

At the end of the treatment, there is a wide vertical space between the posteriors. This is one of the main disadvantages of the Myobrace®. Moreover, a notable drawback of prefabricated appliances is the inadequate space available for premolars to erupt, since these appliances do not allow for the necessary provision to accommodate the spacing required. Therefore, additional research and advancements will be required to elucidate this issue. Depending on the patient's particular needs and compliance, final alignment, in this case, may necessitate a shorter length of treatment with braces to get the best results and create sufficient space for the eruption of upper premolars. The progression of the therapy utilizing fixed orthodontic appliances is shown in Figure 5 for a period of three months. After undergoing fixed orthodontic treatment, it is necessary to use either fixed or removable retainers for a period of 5 years after treatment to maintain the stability of the teeth. This follow-up period is crucial to guarantee that the results achieved from the treatment are maintained throughout time.

## 4. Conclusions

Myofunctional therapy through Myobrace® is an appropriate option for the treatment of Class II division 1 malocclusion in mixed dentition with improper oral habits to achieve a normal occlusion and pleasant facial profile. The findings demonstrated that an early approach in certain situations, while also taking into consideration patient preferences and compliance, is feasible and should be taken into account in the future as a component of treatment planning and related future research.

## 5. Clinical Significance

Class II cases require critical intervention. Whenever, during the mixed dentition stage, Class II skeletal and dental issues are identified, interceptive orthodontic procedures should be performed immediately. The use of myofunctional therapy, such as Myobrace®, in such instances provides the following benefits:

1. Correcting improper myofunctional behaviors by instructing patients to breathe through their nose rather than their mouth.
2. To relax the tongue appropriately on the tip of their hard palate.
3. To train proper swallowing and to maintain closed lips when not eating or speaking.
4. To continue expanding their maxilla and mandible so that they develop correctly, and therefore establishing normal development.

## Figures and Tables

**Table 1:** Lateral cephalometric measurement.

Parameters	Normal (mean ± SD)	Pre-treatment	Pre-debonding
Horizontal skeletal			
SNA (°)	82 ± 2	84	83
SNB (°)	80 ± 2	79	81
ANB (°)	2 ± 2	5	2
Wits appraisal (mm)	1 ± 1	6.5	2.5
Angle of convexity (°)	0 ± 5	6	2
Vertical skeletal			
*Y*-Axis (°)	60 ± 4	60	60
SN-mandibular plane (°)	32 ± 3	29	31
MMPA (°)	27 ± 5	26	28
LAFH (%)	55 ± 2	53	55
Dental			
Interincisal angle (°)	135 ± 10	125	136
U1-palatal plane (°)	109 ± 6	113	108
U1-NA (mm)	4 ± 2	6	5
L1-mandibular plane (°)	90 ± 4	86	90
L1-NB (mm)	4 ± 2	3	4
Soft tissue			
Upper lip—E line (mm)	1±2	5	1
Lower lip—E line (mm)	0 ± 2	−1	0
